# The Potential Regulatory Network of Glutamate Metabolic Pathway Disturbance in Chinese Han Withdrawal Methamphetamine Abusers

**DOI:** 10.3389/fgene.2021.653443

**Published:** 2021-03-23

**Authors:** Sufang Peng, Hang Su, Tianzhen Chen, Xiaotong Li, Jiang Du, Haifeng Jiang, Min Zhao

**Affiliations:** ^1^Shanghai Mental Health Center, Shanghai Jiao Tong University School of Medicine, Shanghai, China; ^2^Shanghai Key Laboratory of Psychotic Disorders, Shanghai, China; ^3^CAS Center for Excellence in Brain Science and Intelligence Technology (CEBSIT), Chinese Academy of Sciences, Shanghai, China

**Keywords:** methamphetamine abuse, metabolomics, bioinformatics, regulatory network, glutamate metabolic pathway

## Abstract

**Objects:**

To explore the long-term influence of methamphetamine abuse on metabolomics character, with gas chromatography-mass spectrometry (GS-MS) technology, and the potential regulatory network using the bioinformatics method.

**Methods:**

Forty withdrawal methamphetamine abusers (WMA) were recruited from Shanghai Gaojing Forced Isolation Detoxification Institute. Forty healthy controls (HC) were recruited from society. GS-MS technology was used to detect metabolic products in serum. A bioinformatics method was used to build a regulatory network. Q-PCR was used to detect the candidate gene expressions, and ELISA was used to detect the regulatory enzyme expressions.

**Results:**

Four pathways were significantly changed in the MA compared to the HC: (1) the arginine synthesis pathway, (2) alanine, aspartic acid and glutamate metabolic pathway, (3) cysteine and methionine metabolic pathway, and (4) the ascorbate and aldarate pathway (enrichment analysis *p* < 0.05, Impactor factor > 0.2). When focusing on the ‘Alanine, aspartate, and glutamate metabolism’ pathway, a regulatory network was established, and the expression of candidate regulatory genes and enzymes was verified. It was found that the expression of *DLG2* (Discs large MAGUK scaffold protein 2), *PLA2G4* (Phospholipase A2 group IVE), *PDE4D* (Phosphodiesterase 4D), *PDE4B* (Phosphodiesterase 4B), and *EPHB2* (Ephrin type-B receptor 2) were significantly different between the two groups (*p* < 0.05), However, after adjusting for age and BMI, only *DLG2, PLA2G4*, and *EPHB*2 remained significant (*p* < 0.05). The expression of enzymes was not significantly different (*p* > 0.05).

**Conclusion:**

Methamphetamine abuse influences the metabolic process in the long term, and *DLG2, PLA2G4*, and *EPHB2* may regulate the glutamate metabolism pathway.

## Introduction

Methamphetamine (METH) is a widely abused addictive psychostimulant, which accounts for a considerable share of global disease burden. METH abuse is increasing rapidly (Yang X. et al., 2018), especially in East and South-East Asia, and Oceania. Long-term abuse of METH causes serious physical and mental damage. Compared to other psychostimulants, METH is better at penetrating the center nervous system (CNS) and has a longer duration of action (Yang X. et al., 2018), making METH more addictive while the damage to the CNS is more serious and persistent. METH addicts often suffer from permanent psychotic symptoms (Mellsop et al., 2019) and cognitive decline, even when deprived from drugs, bringing great difficulties for patients who try to return to society. These symptoms are mostly attributed to METH-induced long-term neurotoxicity and excitotoxicity (Ashok et al., 2017). The neurochemical mechanisms underlying neurotoxicity and excitotoxicity are complex and not well understood. Glutamate, as an important excitatory neurotransmitter is thought to activate various downstream signaling pathways, for example the apoptotic pathway, and eventually produce neurotoxicity (Courtney and Ray, 2014; Yang X. et al., 2018). A previous study found that methamphetamine may result in glutamine system disturbance (Tasic et al., 2017), and glutamate + glutamine in the right inferior frontal cortex is decreased in methamphetamine users (Zheng et al., 2016).

Metabolomics is a potentially powerful tool for understanding the global biological consequences of disease and drug administration. In recent years, with the improvement of metabolomics technology, metabolomics research in psychotic diseases has made great progress (Bortolato et al., 2009; Zhang et al., 2016; Proitsi et al., 2017; Rodríguez Cerdeira et al., 2017). However, in the field of substance addiction, metabolomics research is still in its initial stages. A few studies focus on methamphetamine addiction, using animals as study subjects. Energy metabolic disturbance is believed to be the biochemical base of toxicity and addictive property of MTHE (Zhang et al., 2016). Neurotransmitters in serum or plasma are also disrupted by METH, especially in the glutamate-glutamine-GABA system (Rist et al., 2017). Nevertheless, metabolic character differences between animals and humans still exist. This study uses withdrawal methamphetamine abusers (WMA) as objects and explore the metabolomics characters and possible regulatory mechanisms that may be the basis of the long-term neurotoxicity of methamphetamine.

## Materials and Methods

### Ethics Statement

Informed consent of the participants was obtained after the nature of the procedures used in the study had been fully explained. The study was carried out in accordance with the last version of the Declaration of Helsinki, and the protocol was approved by the Ethics Committee of Shanghai Mental Health Center (SMHC IRB 2016-11R). All participants were identified using numbers, instead of names.

### Research Participants

Forty male WMA were recruited from Shanghai Gaojing Forced Isolation Detoxification Institute in July 2017, and 40 male healthy controls were recruited from general society. The entire sample consisted of a Chinses Han population. The WMA accepted assessment from two senior psychiatric doctors and met the diagnosis criterion for methamphetamine abuse disorder according to the Diagnostic and Statistical Manual of mental disorders, fifth edition (DSM-5). Participants did not have the opportunity to use any illicit substance during the last 2 months. They completed a self-report form that included basic demographic information and accepted an interview of Addiction Severity Index (ASI). Exclusion criteria for both groups were: (1) history of craniocerebral trauma or epileptic seizure; (2) metal implants in the brain or an implanted pacemaker; (3) history of major physical diseases, organic mental disorders, and severe mental disorders. People in the control group did not have any history of illegal substance abuse. Venous blood was collected at 7:30–8:00 am before breakfast.

### Gas Chromatography – Mass Spectroscopy (GC-MS)

After standing at room temperature for 30 min, venous blood was centrifuged at 4°C, 3000*g*, for 15 min. The upper layer of the serum was collected. Sample preparation, GC-MS experimental process, and GC-MS data processing were carried out using previously published methods (Morris et al., 2012; Li et al., 2014). Compound identification was performed by comparing the retention indices and mass spectral data with those previously generated from reference standards of known structures present in the JiaLib metabolite database using the proprietary software XploreMET. The current JiaLib comprises over 1,200 mammalian metabolites over a 15-year accumulation. The reference chemicals present in JiaLib were commercially purchased from Sigma-Aldrich (St. Louis, MO, United States), Santa Cruz (Dallas, TX, United States), Nu-Chek Prep (Elysian, MN, United States), and synthesized in the laboratory. XploreMET adopted a specific algorithm hypergeometric, which can enrich the information of metabolites, weight the contribution of metabolites, and focus on the key metabolic network. The *P* and impact value was used to measure the importance of metabolic pathway.

### Bioinformatics Analysis

Based on the results of untargeted metabolomics, a glutamate metabolism pathway was noticed. Glutamate is a very important excitatory neurotransmitter, and a previous study has confirmed that glutamate + glutamine in the right inferior frontal cortex was decreased in methamphetamine users (Zheng et al., 2016). In serum or plasma, the glutamate-glutamine-GABA system was also disrupted by METH (Rist et al., 2017). So, we chose the ‘alanine, aspartic acid and glutamate metabolic pathway’ to build the regulatory network.

The four differential expressed metabolites were treated as candidate metabolites. We collected 133 risk genes from previous genome-wide association studies (Heikkinen et al., 2018; Irwin et al., 2018). The molecular network was constructed by connecting candidate metabolites, enzymes, intermediate proteins, and risk genes. First, we investigated the relative enzymes linked to these candidate metabolic compounds according to the KEGG database and the Human Metabolome Database (HMDB). To further explore correlation networks linked to the relative enzymes and risk genes, we searched the functional proteins network database (STRING v10.5) setting the requirement that the score must be more than 700 and that no more than one intermediate protein must be present between the relative enzyme and risk gene. Finally, we built a network including four enzymes: *ASNS* (Asparagine synthetase), *AGT* (Angiotensinogen), *GAD1* (Glutamate decarboxylase 1), and *GAD2* (Glutamate decarboxylase 2); 13 genes: *DLG2* (Discs large MAGUK scaffold protein 2), *NRG1* (Neuregulin 1), *PLA2G4E* (Phospholipase A2 group IVE), *STX8* (Syntaxin 8), *NEDD4L* (NEDD4 like E3 ubiquitin protein ligase), *PRKG1* (Protein kinase cGMP-dependent 1), *PDE4D* (Phosphodiesterase 4D), *PDE4B* (Phosphodiesterase 4B), *SERPINA5* (Serpin family A member 5), *PDE6C* (Phosphodiesterase 6C), *CD55* (CD55 molecule), *EPHB2* (Ephrin type-B receptor 2), and *RAPGEF5* (Rap guanine nucleotide exchange factor 5), and several intermediate proteins.

### RNA Extraction and Gene Expression Analysis

RNA was extracted from the serum samples with Trizol reagent (Invitrogen, Carlsbad, CA, United States) according to the previous protocol (Gan et al., 2018), and stored at −80°C for subsequent cDNA synthesis. Following RNA quantitation, 1ug of total RNA was used for cDNA synthesis (5 × All-In-One RT Master Mix, ABM, Canada), and the quantitative gene expression analysis was performed using TB Green Fast qPCR Mix (TaKaRa, Shiga, Japan), and Lightcycle^®^ 96 real-time PCR machine (Roche Diagnostics GmbH, Germany). Primers are listed in Table 1. *GAPDH* was used as a housekeeper gene. The relative gene expression was calculated using the 2^−△CT^ method (Kohno et al., 2016). The CT values of duplicates were averaged for the final analysis.

**TABLE 1:** Primers used in Q-PCR.

**gene**	**Primers (F: Forward; R: Reverse)**
GAPDH	F AGGGCTGCTTTTAACTCTGGT R CCCCACTTGATTTTGGAGGGA
DLG2	F CCCAATGGGATGGCAGACTT R AGTATTCCCACCTCCCTCCC
NRG1	F GCAACTTTGTTTCCCGGGTC R TTGGGGGCAAAAGTCACACT
PLA2G4E	F AATCAGTGCTCCCTTGAGCC R GCATTACCTGGATGGGGACC
STX8	F CCCCTGGTTCTCCACATACG R TCTGTCTTCGGTCCCCTTCA
NEDD4L	F AGGGTTAGCTTCCTGTTGGC R TGGTACGGGGTTGAGAATGC
PRKG1	F ACTTGGTGCACCTCTCAACA R AGTCATTGCCAAGGTCCCAG
PDE4D	F TCTTACAGCCCACGGGGATA R AGGCAGAATCAACCCATGCT
PDE4B	F GTGTCGTTCACCGTGAGAGT R CGGGGTGAAGAGAGGAGGTA
CD55	F CATCTTTCCTTCGGGTTGGC R CACCATCAACACCCCTGGTT
EPHB2	F AGTCTGGGGAGGGACTCATC R CTGTGTGGCCATGGAAGCTA
RAPGEF5	F GGGCTTTCACTGCTACCCAT R GATTGGCTGCGCCATTTAGG
SERPINA5	F GCTATGGCCCATCTGTATGCT R TTCCCCAAGGCACCTGTATG
PDE6C	F TTTTGGAGAGGCACCACCTG R AACTGTTTCAAACTGCCGCT

### Elisa

The serum levels of *AGT, GAD1, GAD2*, and *ASNS* were assessed using an ELISA kit (catalog number SEC904Hu, Cloud-Clone Corp, Shanghai, China for *GAD1*, and catalog numbers MBS726474, MBS2501551, MBS090144; Mybiosource, Southern California, San Diego, United States, for *AGT, GAD2*, and *ASNS*, respectively).

All assays were performed according to the manufacturer’s directions and were performed in duplicate. The average value of the duplicates was used as the final value.

### Statistical Analysis

General data was presented as mean and standard error of the mean (Mean ± SD), and analyzed using SSPSS 22.0. Data was analyzed using the student *t* test, a was 0.05 and Cohen’s d was used to calculate the effect size of the *t* test. 2^–△CT^ was used to calculate the relative expression of genes, and the expression ratio of the WMA group relative to the control group was calculated as previously described (Kohno et al., 2016).

Metabolomics data was managed with the XploreMET system (v3.0, Metabo-Profile, Shanghai, China), and R was used to analyze the data. The NIST 2017 database was used for metabolite certification and Metaboanalyst 3.0 was used for the metabolic pathway analysis. Partial least squares discriminant analysis (PSL-DA) was performed, and a *t* test was used after log transformation. Differential expressed metabolites were screened according to fold change (FC) and *p* value. FC > 1. 5 and *p* < 0. 05 showed that the metabolite was significantly differentially expressed. Based on the selected differential metabolites, a pathway analysis was performed using a hypergeometric test and KEGG pathways (Yang W. et al., 2018) and the *p* values were calculated as follows:

$$\begin{array}{c} P_{p\,t\,w}(Ptw_{H\,i\,t\,s}|Total_{A\,l\,l},Ptw_{A\,l\,l},Total_{H\,i\,t\,s}\\ \frac{\left(\begin{array}{c} P\,t\,w_{A\,l\,l}\\ P\,t\,w_{H\,i\,t\,s} \end{array}\right)\,\left(\begin{array}{c} T\,o\,t\,a\,l_{A\,l\,l}-P\,t\,w_{A\,l\,l}\\ T\,o\,t\,a\,l_{H\,i\,t\,s}-P\,t\,w_{H\,i\,t\,s} \end{array}\right)}{\left(\begin{array}{c} T\,o\,t\,a\,l_{A\,l\,l}\\ T\,o\,t\,a\,l_{H\,i\,t\,s} \end{array}\right)} \end{array}$$

Ptw_Hits_ is the hit number of differential metabolites in a certain metabolic pathway. Total_All_ is the number of metabolites in all pathways of a certain species, such as Homo sapiens. Ptw_All_ is the total number of metabolites in a certain pathway and Total_Hits_ is the hit number of differential metabolites in all pathways. A significant *p* value means significantly changed metabolic pathway activities that need to be focused on. For multiple tests, a false discovery rate (FDR) was applied to correct the raw *p* values.

## Results

### Demography Characteristic

The demographic characteristic are shown in Table 2. Age, BMI, and education level between the two groups were not significantly different. However, WMA individuals have lower marriage rates and higher unemployment (*p* < 0.05).

**TABLE 2:** Demographic characteristics of subjects.

	**WMA (Mean ± SD)**	**Control (Mean ± SD)**	***t* value**	***p* value**
Age (years)	34.02 ± 6.82	32.02 ± 8.57	1.13	0.260
BMI (kg/m2)	24.44 ± 3.64	23.54 ± 3.23	1.14	0.260
Education (years)	5.95 ± 3.55	6.97 ± 3.14	1.14	0.256
Marital status			16.537	0.000
Married	9 (23.1%)	20 (52.6%)		
Divorced/Separated	15 (38.4%)	1 (2.6%)		
Unmarried	15 (38.4%)	17 (44.7%)		
Employment			8.271	0.004
Unemployed	18 (46.1%)	6 (15.8%)		
The age began to abuse (years)	25.20 ± 7.52			
Withdrawal time (month)	3.20 ± 1.32			
Duration of abuse (years)	6.51 ± 3.26			
Average dosage (g)	0.64 ± 0.35			
**Frequency**				
Everyday	24 (60.0%)			
3–5 times/week	12 (30.0%)			
1 time/week	2 (5.0%)			
1–3 times/month	2 (5.0%)			

### Metabolomics Profile and Pathways

Thirty-five differentially expressed metabolites were screened by *t* test (*p* < 0.05), in which 21 metabolites could be found by searching metabolic pathways in the KEGG information database (as shown in Table 3). Since age and BMI may influence the metabolic function, we adjusted age and BMI. After that, there were 19 metabolites differentially expressed in two groups, including two kinds of alcohols, seven kinds of amino acids, one kind of carbohydrate, one kind of fatty acid, three kinds of nucleotides, and five kinds of organic acids.

**TABLE 3:** Differentially expressed metabolites in pathways.

**Metabolites species**	**Metabolites**	**Metabolic pathways**	**FC value**	**Before adjusting**	**After adjusting**	**Cohen’s d**
				***p* value**	***p* value**	
Alcohols	Glycerin	Galactose metabolism, glyceride metabolism	0.6	0.000	0.000	0.898
Alcohols	Inositol	Galactose metabolism, inositol metabolism, inositol phosphate metabolism, phosphatidylinositol phosphate metabolism	0.7	0.001	0.001	0.813
Amino acids	1-Methylhistidine	Histidine metabolism	1.4	0.001	0.001	–0.802
Amino acids	L- Aspartic acid	Ammonia recovery, arginine and proline metabolism, aspartic acid metabolism, B-alanine metabolism, malate-aspartic acid shuttle, transcription/translation, urea cycle	1.5	0.001	0.002	–0.761
Amino acids	Homocysteine	Betaine metabolism, catecholamine biosynthesis, glycine and serine metabolism, homocysteine degradation, methionine metabolism	1.4	0.004	0.004	–0.692
Amino acids	L- Methionine	Betaine metabolism, glycine and serine metabolism, methionine metabolism, spermidine and spermine biosynthesis, transcription/translation	0.9	0.005	0.009	0.658
Amino acids	L- Glutamic acid	Amino sugar metabolism, ammonia recovery, glutamate metabolism, phenyl acetate metabolism, purine metabolism, pyrimidine metabolism, transcription/translation, urea cycle	1.1	0.011	0.015	–0.597
Amino acids	Ornithine	Metabolism of arginine and proline, metabolism of glycine and serine, biosynthesis of spermine and spermine, urea cycle	1.3	0.013	0.020	–0.582
Amino acids	L- Cysteine	Cysteine Metabolism, Glutathione Metabolism, Glycine and Serine Metabolism, Methionine Metabolism, Pantothenate and CoA Biosynthesis, Tauurine and Tauurine Metabolism, Transcription/Translation	0.9	0.028	0.016	0.517
Amino acids	L- Isoleucine	Degradation, transcription/translation of valine, leucine and isoleucine	1.1	0.029	0.088	–0.512
Amino acids	L- Proline	Arginine and proline metabolism, transcription/translation	1.2	0.032	0.068	–0.502
Carbohydrate	Sorbitol	Fructose and mannose degradation, galactose metabolism	0.9	0.025	0.027	0.525
Fatty acids	Linoleic acid	α-linolenic acid and linoleic acid metabolism	0.8	0.007	0.008	0.643
Nucleotides	Hypoxanthine	Purine metabolism	1.4	0.000	0.000	–1.046
Nucleotides	Xanthine	Purine metabolism	1.2	0.000	0.000	–0.900
Nucleotides	Guanosine hydrate	Purine metabolism	0.7	0.005	0.006	0.658
Organic acids	Glyceric acid	Glyceride metabolism, glycine and serine metabolism	0.7	0.000	0.000	1.023
Organic acids	Pyruvic acid	Alanine metabolism, amino glucose metabolism, ammonia recovery, tricarboxylic acid cycle, cysteine metabolism, gluconeogenesis, glucose-alanine cycle, glycine and serine metabolism, glycolysis, pyruvic aldehyde degradation, pyruvate metabolism, acetyl mitochondrial transfer, urea cycle	0.6	0.000	0.000	0.872
Organic acids	Vanillylmandelic_acid	Tyrosine metabolism	0.8	0.002	0.002	0.744
Organic acids	Hydroxypropionic_acid	Propionic acid metabolism	0.9	0.033	0.027	0.497
Organic acids	Fumaric acid	Arginine and proline metabolism, aspartic acid metabolism, citric acid cycle, mitochondrial electron transport chain, phenylalanine and tyrosine metabolism, tyrosine metabolism, urea cycle	0.7	0.037	0.047	0.488

**FIGURE 1:**
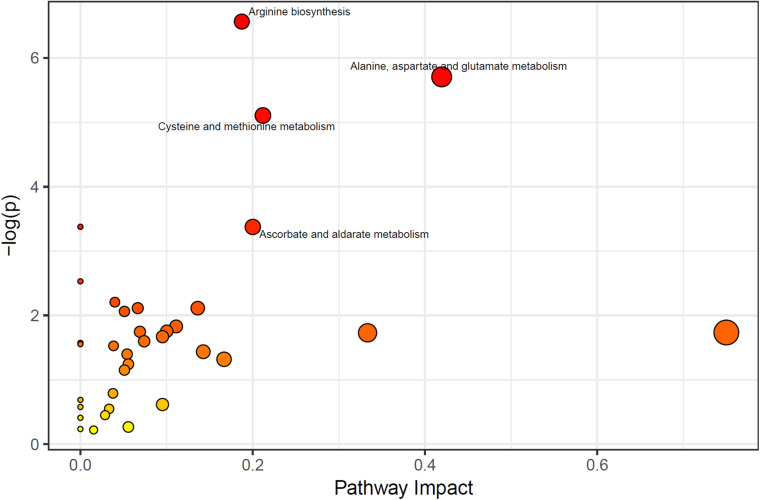
Significant pathways (the pathways which *p* < 0.05, and Impactor factor > 0.02 were marked out).

According to the results of the Metabolic Pathway Enrichment Analysis (MPEA), four pathways were found to be significantly changed; (1) arginine synthesis pathway, (2) alanine, aspartic acid and glutamate metabolic pathway, (3) cysteine and methionine metabolic pathways, and (4) ascorbate and aldarate pathway (enrichment analysis *p* < 0.05,Impactor factor > 0.2) (Figure 1).

### Glutamine Metabolites Regulatory Network

**FIGURE 2:**
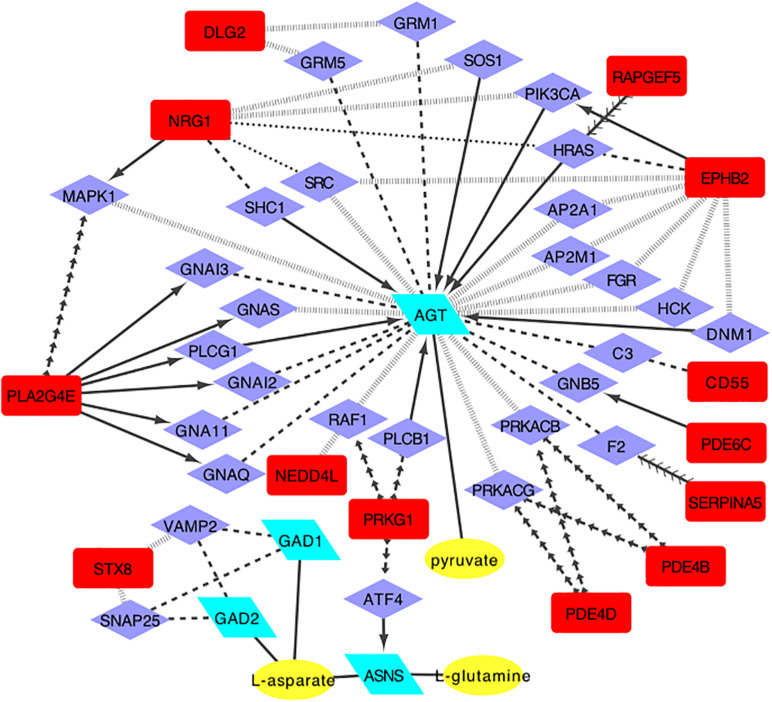
The regulatory network model for alanine, aspartic acid and glutamate metabolism pathway(enzyme, posttranslational modification, activate, inhibition, catalysis, reaction, and combine. Yellow oval, metabolites of glutamate pathway; Green parallelogram, regulatory enzymes; Purple diamond, intermediate proteins; Red square, candidate genes).

Previous research found that methamphetamine may result in glutamine system disturbance (Tasic et al., 2017), either in the brain and serum or in plasma (Zheng et al., 2016; Rist et al., 2017). This study also found a change in the glutamate pathway. We therefore focused on the ‘Alanine, aspartate, and glutamate metabolism’ pathway, and based on the bioinformatics method, the regulatory network was built. As shown in Figure 2, we found three metabolites (pyruvate, L-aspartate, and L-glutamine), which can be regulated by four enzymes, and which can be related to 13 candidate genes. Each candidate gene influences the regulatory enzyme through one intermediate protein (Figure 2).

### Candidate Gene Expression

According to the regulatory network, we used Q-PCR to verify the 13 candidate genes expressed. 2^–△^^CT^ was used to calculate the relative expression of genes (Kohno et al., 2016) (Table 4). *DLG2, PLA2G4, PDE4D, PDE4B*, and *EPHB2* were significantly differentially expressed between the two groups (*p* < 0.05). However, after adjusting for age and BMI, only *DLG2, PLA2G4*, and *EPHB2* remained significant (*p* < 0.05). *STX8, RAPGEF5*, and *PDE6C* were not detected because of their low expression.

**TABLE 4:** Expression of candidate genes.

	**WMA**	**Control**	**Before adjusting**		**After adjusting**		**Cohe^n′^sd**
			***t* value**	***p* value**	***t* value**	***p* value**	
DLG2	0.250 ± 0.104	0.190 ± 0.083	2.804	0.006	–2.348	0.022	0.638
NRG1	0.220 ± 0.071	0.210 ± 0.066	1.207	0.231	–1.002	0.320	0.146
PLA2G4	0.050 ± 0.028	0.020 ± 0.024	4.260	0.000	–3.672	0.000	1.150
STX8	–	–	–	–	–	–	–
NEDD4L	0.140 ± 0.041	0.140 ± 0.053	–0.532	0.596	0.528	0.599	–0.101
PDE4D	0.140 ± 0.038	0.120 ± 0.050	2.007	0.048	–1.665	0.100	0.450
PDE4B	0.170 ± 0.039	0.150 ± 0.040	2.191	0.032	–1.722	0.090	0.506
CD55	0.110 ± 0.032	0.100 ± 0.036	0.912	0.365	–0.609	0.545	0.294
EPHB2	0.280 ± 0.137	0.190 ± 0.065	3.731	0.000	–3.324	0.001	0.839
RAPGEF5	–	–	–	–	–	–	–
SERPINA5	0.220 ± 0.076	0.190 ± 0.052	1.904	0.061	–1.639	0.106	0.461
PDE6C	–	–	–	–	–	–	–

**FIGURE 3:**
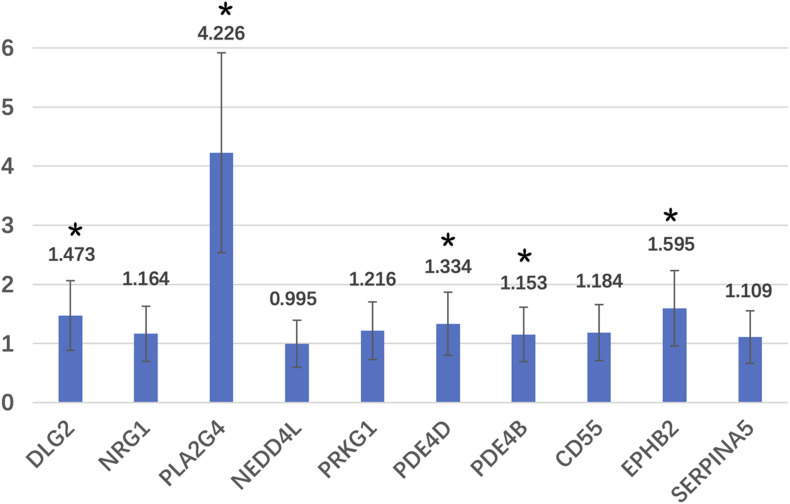
Relative expression fold of candidate genes. *Means that these genes are significantly different expressed in two groups.

For the *DLG2, PLA2G4, PDE4D, PDE4B*, and *EPHB2* genes, the expression in the WMA group was 1.473, 4.226, 1.334, 1.153, and 1.595 times higher than that in control group, respectively (Figure 3).

### Regulatory Enzyme Expression

ELISA was used to detect the regulatory enzyme expression. As Table 5 shows, no significant difference was found between the two groups (*p* > 0.05).

**TABLE 5:** Expression of candidate enzymes.

	**WMA**	**Control**	***t* value**	***p* value**	**Cohen’s d**
ASNS	2.28 ± 1.65	2.21 ± 1.44	0.18	0.86	0.045
AGT	3.31 ± 1.23	3.52 ± 1.64	–0.63	0.53	–0.145
GAD1	0.49 ± 0.13	0.50 ± 0.12	–0.34	0.74	–0.080
GAD2	3.88 ± 0.92	3.71 ± 1.56	0.59	0.54	0.133

## Discussion

This study found that peripheral metabolites of WMA patients were mainly changed in amino acid metabolism. The most obvious pathways were; (1) the arginine synthesis pathway, (2) the alanine, aspartic acid and glutamate metabolic pathways, (3) the cysteine and methionine metabolic pathways, and (4) the purine metabolic pathway.

Previous studies on methamphetamine addicted animals found that abnormal energy metabolism was the biochemical basis of toxicity and addiction (Cservenka and Ray, 2017; Moallem et al., 2018). An increase in energy metabolism was found in the serum of amphetamine-administered rats. After 5 consecutive days of administration, the difference between the administration and control group decreased (Moallem et al., 2018). After high dose, high frequency, or long time administration, the energy reserve may be depleted (Moallem et al., 2018). The patients in this study were basically deprived from METH for about 2 months. This may explain why abnormal energy metabolism was not observed—the energy reserves may have been consumed.

In addition, amphetamines cause serum/plasma neurotransmitter disturbances mainly in the glutamate-glutamine-GABA system (Moallem et al., 2018). Glutamate-Glutamine disturbance was observed both in withdrawal animals and in the WMA patients (Shima et al., 2011; Chen et al., 2015; Su et al., 2018), and this disturbance may be related to a continuous search for drugs and the high relapse risk (Zheng et al., 2014). Glutamate, aspartic acid, and methionine are elevated in rat neuroblastoma cells 48 h after methamphetamine treatment (Bu et al., 2013). These studies showed that the effect of methamphetamine on the glutamate system could last for a long time. In this study, the “alanine, aspartic acid, and glutamate pathway” was found to be abnormal, and the expression of related metabolites was significantly changed, which is consistent with previous studies.

We chose the glutamate metabolic pathway and used bioinformatics methods to establish a regulatory network model, which predicts that *GAD1, GAD2, ASNS*, and *AGT* may directly regulate the glutamate pathway metabolites- pyruvate, L-aspartate, L-glutamine. The regulatory enzyme is regulated by *DLG2, NRG1, PLA2G4E, STX8, NEDD4L, PRKG1, PDE4D, PDE4B, SERPINA5, PDE6C, CD55, EPHB2*, and *RAPGEF5* genes only through one intermediate protein. We verified the expression of the candidate genes and enzymes, and finally found five regulatory genes that were differentially expressed in the two groups, namely *DLG2, PLA2G4, PDE4D, PDE4B*, and *EPHB2* genes. After adjusting for age and BMI, only *DLG2, PLA2G4*, and *EPHB2* remained significant.

*DLG2* plays a very important role in synaptic development and stability of plasticity (McClay et al., 2013; Su et al., 2020). The function of the *DLG2* gene is associated with complex cognitive and learning tasks (Parsegian and See, 2014), and is also associated with developmental and intellectual disorders (Christopher et al., 2007) such as Parkinson’s disease (Darna et al., 2015). The *DLG2* gene polymorphism was relevant for methamphetamine addiction (Irwin et al., 2018). This study also found that the expression of the *DLG2* gene was up-regulated in WMA subjects. There was a long-term cognitive impairment in methamphetamine addicted populations (Crocker et al., 2014), as well as dysfunction of the glutamate system (Maker et al., 2018). The *DLG2* gene may play an important role in the development, plasticity, and stability of glutamatergic synapses and may further affect the cognitive function of WMA patients.

The protein coded by the *PLA2G4E* gene is involved in the regulation of membrane-mediated transport and affects phospholipase activity (Zhu et al., 2016). *PLA2G4E* is strongly expressed in neurons and regulates endogenous cannabinoids, by mobilizing intracellular calcium (Zhu et al., 2016). The endogenous cannabinoid system also regulates the function of the glutamate system. Cannabinoid 1 receptors affect glutamatergic and GABAergic regulation of anxiety and fear responses (Zheng et al., 2011; Nithianantharajah et al., 2013). The PLA*2G4E* gene expression changed the most in this study, reaching 4.226 times that of the control group. That may influence cannabinoid, glutamate, and GABA system functions.

The *PDE4D* and *PDE4B* genes belong to the phosphodiesterase (PDE family) superfamily, and play an important role in the degradation of cAMP (Reggiani et al., 2017). Since cAMP is an important second messenger, *PDE4D/4B* may play a role in signal transduction by regulating cAMP concentration. *PDE4D*/*4B* is widely expressed in the human brain. Previous studies have shown that *PDE4D/4B* is associated with neuropsychiatric disorders such as depression, schizophrenia (Wu et al., 2018), methamphetamine addiction (Irwin et al., 2018), alcohol-induced neuroinflammation (Ikeda et al., 2013), and stroke (Zhong et al., 2016). Since signal transduction of neurotransmitters in the brain, such as dopamine, glutamate, and GABA, etc., requires the participation of second messenger cAMP, some researchers believe that the phosphodiesterase family (PDE) may be used as an important drug target for schizophrenia and affective disorder (Wu et al., 2018). This study found that the expression of *PDE4D/4B* in the peripheral blood of WMA patients was significantly higher than that of the control group. However, after adjusting for age and BMI, this difference was no longer significant. The expression ratios of the WMA group compared to the control group were also close to 1, showing that these two genes may not be important regulatory genes in the glutamine system of WMA.

The *EPHB2* gene encodes Ephrin type-B receptor 2, which is a tyrosine kinase receptor involved in triggering and maturation. In mature brains, *EPHB2* is highly abundant in large dendritic axes and in the frontal cortex and dendritic spines in the hippocampus, and is also involved in the regulation of glutamate receptor distribution and excitatory synapse formation (Su et al., 2017). *EPHB2* also regulates synaptic plasticity and leads to anxiety behavior (Capestrano et al., 2014) and reversal of cognitive deficits (Ruehle et al., 2012). *EPHB2* reverses cognitive dysfunction in the Alzheimer’s disease model, possibly related with glutamate receptor-NMDA’s function (N-methyl-D-aspartic acid receptor), and EPHB2 is a key regulator of synaptic localization of NMDA receptors (Hu et al., 2017). Knockout *EPHB2* induces depression-like behavior and memory impairment in mice, which is mediated by NMDA receptor subtypes (Zhen et al., 2018). This study found a significant increase of the EPHB2 gene in WMA patients, which elicits that an abnormal expression of the EPHB2 gene may be a potential mechanism of glutamate system dysfunction and emotional or cognitive symptoms of methamphetamine addicts.

The expression of candidate regulatory enzymes was not differentially expressed between two groups. However, the activity of enzymes was not detected, which may influence the metabolites. Another complex regulatory mechanism could not be excluded.

## Conclusion

MA use has a long-term and extensive impact on the human body, which can be reflected by the peripheral metabolites. The glutamate system metabolic disturbance may be regulated by the genes that are associated with multiple pathophysiological processes such as synaptic plasticity, signal transduction, cAMP degradation, phosphorylation, and synaptic localization, which may be related to clinical symptoms of withdrawal MA, such as long-term cognitive impairment, anxiety and fear responses, and so on.

There were some limitations in this study. First, only one time point was selected for the exploration of serum metabolomics in the addiction state of methamphetamine. If long-term follow-up can be performed during the acute phase and withdrawal period, the changes in metabolomics characteristics over time will be observed and the results will be more systematic. Second, the exploration of the mechanism is limited to biochemical and gene regulation mechanisms, and if it can be combined with the analysis of central neurotransmitter changes, it will be more in-depth. Third, the enzyme activity was not detected. Fourth, the sample size was limited, and all the enrolled participants were from the Shanghai area only. The conclusion can therefore only be extended to Shanghai Han nationality. A multi-center collaboration may therefore facilitate further discoveries.

## Data Availability Statement

The raw data supporting the conclusions of this article will be made available by the authors.

## Ethics Statement

The studies involving human participants were reviewed and approved by Ethics Committee of Shanghai Mental Health Center. The patients/participants provided their written informed consent to participate in this study.

## Author Contributions

SP: conceptualization, data curation, formal analysis, methodology, writing – original draft, review, and editing. HS: investigation, project administration, and supervision. TC: investigation and project administration. XL: investigation and project administration. JD: conceptualization, methodology, project administration, resources, and supervision. HJ: conceptualization, methodology, project administration, resources, and supervision. MZ: conceptualization, funding acquisition, methodology, resources, supervision, and writing – review and editing. All authors contributed to the article and approved the submitted version.

## Conflict of Interest

The authors declare that the research was conducted in the absence of any commercial or financial relationships that could be construed as a potential conflict of interest.
